# The Novel 10-Item Asthma Prediction Tool: External Validation in the German MAS Birth Cohort

**DOI:** 10.1371/journal.pone.0115852

**Published:** 2014-12-23

**Authors:** Linus B. Grabenhenrich, Andreas Reich, Felix Fischer, Fred Zepp, Johannes Forster, Antje Schuster, Carl-Peter Bauer, Renate L. Bergmann, Karl E. Bergmann, Ulrich Wahn, Thomas Keil, Susanne Lau

**Affiliations:** 1 Institute for Social Medicine, Epidemiology and Health Economics, Charité – Universitätsmedizin Berlin, Berlin, Germany; 2 Department for Psychosomatic Medicine, Clinic for Internal Medicine, Charité – Universitätsmedizin Berlin, Berlin, Germany; 3 Centre for Paediatric and Adolescent Medicine, University Medical Centre Mainz, Mainz, Germany; 4 St Josefs Hospital, Department of Paediatrics, Freiburg, Germany; 5 Department of Paediatrics, Heinrich-Heine-University, Dusseldorf, Germany; 6 Department of Paediatrics, Technical University of Munich, Munich, Germany; 7 Department of Obstetrics, Charité – Universitätsmedizin Berlin, Berlin, Germany; 8 Department of Paediatric Pneumology and Immunology, Charité – Universitätsmedizin Berlin, Berlin, Germany; 9 Institute of Clinical Epidemiology and Biometry, University of Würzburg, Würzburg, Germany; University Medical Center Rotterdam, Netherlands

## Abstract

**Background:**

A novel non-invasive asthma prediction tool from the Leicester Cohort, UK, forecasts asthma at age 8 years based on 10 predictors assessed in early childhood, including current respiratory symptoms, eczema, and parental history of asthma.

**Objective:**

We aimed to externally validate the proposed asthma prediction method in a German birth cohort.

**Methods:**

The MAS-90 study (Multicentre Allergy Study) recorded details on allergic diseases prospectively in about yearly follow-up assessments up to age 20 years in a cohort of 1,314 children born 1990. We replicated the scoring method from the Leicester cohort and assessed prediction, performance and discrimination. The primary outcome was defined as the combination of parent-reported wheeze and asthma drugs (both in last 12 months) at age 8. Sensitivity analyses assessed model performance for outcomes related to asthma up to age 20 years.

**Results:**

For 140 children parents reported current wheeze or cough at age 3 years. Score distribution and frequencies of later asthma resembled the Leicester cohort: 9% vs. 16% (MAS-90 vs. Leicester) of children at low risk at 3 years had asthma at 8 years, at medium risk 45% vs. 48%. Performance of the asthma prediction tool in the MAS-90 cohort was similar (Brier score 0.22 vs. 0.23) and discrimination slightly better than in the original cohort (area under the curve, AUC 0.83 vs. 0.78). Prediction and discrimination were robust against changes of inclusion criteria, scoring and outcome definitions. The secondary outcome ‘physicians’ diagnosed asthma at 20 years' showed the highest discrimination (AUC 0.89).

**Conclusion:**

The novel asthma prediction tool from the Leicester cohort, UK, performed well in another population, a German birth cohort, supporting its use and further development as a simple aid to predict asthma risk in clinical settings.

## Introduction

Our understanding of modifiable and non-modifiable determinants influencing the onset and development of asthma in adolescence advanced in recent years, but it has not lead to improved prevention strategies [1]–[6].

While primary prevention is lacking, the knowledge about essential parameters gathered in research can at least be used to predict future asthma in the clinical setting. Screening children in preschool age to identify those with a high probability of later asthma opens the opportunity for interventions aiming to slow or stop progression or modify disease severity at pre-clinical stages. Prediction or early diagnosis allows for learning about the immunologic processes at work before obvious symptoms occur.

For example, crude definitions of suggestive symptoms and aspects of patient's history are already used in current guidelines to evaluate risk and to target trial treatment (eg, inhaled corticosteroids, [7], [8]). Several formalized prediction algorithms have been proposed to quantify the information content of symptoms, behavioural patterns and heredity for estimating probabilities of later asthma [9]–[12].

In a recent issue of the *Journal of Allergy and Clinical Immunology*, Pescatore et al. [13] introduced a new 10-item asthma prediction tool, based mainly on indicative symptoms, and accounting for age, sex, comorbid eczema and parents' history of asthma/bronchitis. Unlike prior studies, final selection of variables/predictors was run by a LASSO-penalized regression model, homing on fewer factors and higher external validity (Least Absolute Shrinkage and Selection Operator).

None of these scoring systems is widely used in primary care [14]. This is mainly due to the lack of external validation and impact studies, requisites for a general recommendation of such tools [15]. The predictive performance outside the population from which the tool was developed is usually lower than estimated from internal validation, both in retrospective as well as prospective external validation studies [16]–[19]. Pescatore et al. validated the new asthma prediction tool in the cohort used for development itself [13], [20], which now requires replication in other populations [21] as the most rigorous assessment of a model's validity [22].

Our aim was therefore to externally validate Pescatore's asthma prediction tool by estimating measures of discrimination, calibration and performance in the MAS-90 birth cohort [23], [24], supported by sensitivity analyses considering different inclusion criteria for validation sample, scoring items, and outcome definitions, and assessing asthma phenotypes up to 20 years.

## Methods

For external validation of the novel asthma prediction tool we retrospectively applied the suggested scoring to a subsample of the MAS-90 birth cohort. Exploring the robustness of the primary model and accounting for differences in data collection, we reiterated analyses with various definitions of inclusion, scoring and outcome criteria as described below.

### Setting

From all 7,609 children born during 1990 in 6 participating hospitals across Germany, a population-based birth cohort was recruited (n = 1,314, The German Multicentre Allergy Study, MAS-90). Newborns to allergic parents, based on history or positive Immunoglobulin E screening were partly oversampled (19% in all children vs. 38% in the recruited cohort, details in [23], [25], [26]). Development of allergic diseases including asthma as well as information on living environment and lifestyle were traced at nineteen time points up to age 20 years through interviews, questionnaires, clinical investigations including blood sampling and assessment of lung function, achieving a long-term response of 72% at 20 years. The project was approved by local institutional review boards (ethics committees Charité – Universitätsmedizin Berlin; Technical University Munich, Faculty of Medicine; Landesärztekammer Rheinland-Pfalz; University Medical Hospital Freiburg). All parents and later all adult participants provided written informed consent for data collection and analysis.

### Population and inclusion criteria

The sample used for external validation was limited to children who participated in assessments at 3 and 8 years (7^th^ and 12^th^ follow-up). Resembling the development cohort [13], our primary inclusion criteria were further restricted to participants reporting wheeze (‘Has your child had wheezing or whistling in the chest in the last 12 months?’) or cough (‘In the last 12 months, has your child had a dry cough at night, apart from a cough associated with a cold or a chest infection?’) in 3 years' interview. For sensitivity analyses, secondary sample inclusion criteria comprise the whole initial sample irrespective of symptoms at 3 years, and those reporting either wheeze or cough only.

### Scoring variables

Six of the 10 items of the original asthma prediction tool refer to parent-reported symptoms (questions 3–8). For the (primary) scoring definition we manually identified corresponding items from the interview at 3 years in the MAS-90 cohort. Child's sex was documented at recruitment (baseline assessment), age was calculated from date of birth and interview (questions 1, 2). Information on comorbid eczema and parents' allergies were derived from the interview at 3 years (questions 9, 10). Several secondary scoring approaches were assessed. Questions on eczema and parents' allergies asked at 3 years of age covered only the previous 12 months, and were thus complemented with data from earlier follow-ups and baseline. Secondly, all missing information (answer ‘Always’ in question 6, question 8 entirely) was imputed at random. Finally, actual scores were shuffled at random between study participants, giving reference measures of discrimination and performance.

### Outcomes

As used for model development, parent-reported current wheeze (‘Has your child had wheezing or whistling in the chest in the last 12 months?’) in combination with use of asthma drugs (‘Did your child take any drugs against asthma during the last 12 months?’) both at age 8 years was defined as the primary outcome.

As secondary outcomes, wheeze or asthma drugs only, and a physician's diagnosis of asthma were used as reported at the eight-year-follow-up. Another set of secondary outcome definitions used information collected in the MAS-90 cohort in later assessments up to age 20 years. For these outcome definitions asthma was defined as satisfying 2 of the following 3 criteria at any follow-up from age three years and above: physician's diagnosis of asthma ever; asthma drugs in last 12 months; any indicative symptom in last 12 months (wheezing, shortness of breath, dry cough at night). Allergic asthma further included a positive serum specific immunoglobulin E≥0.35 kU/l (kilo Units per litre) to at least one regularly assessed aero-allergen (dust mite, dog, cat, birch, timothy), determined at nine time points (ImmunoCAP – Phadia GmbH, Freiburg, Germany). Lung function at 20 years was assessed through escalating-dose Methacholine challenge, a≥20% drop of FEV_1_ (Forced Expiratory Volume in 1 second) from baseline was considered as increased airway responsiveness.

### Statistical methods

Data management, data cleaning and statistical evaluation was carried out using the SAS system (version 9.3, SAS Institute Inc., Cary NC, USA). Children with missing information on one of the two items used for inclusion or one of the two used for the primary outcome definition were excluded from this analysis. Missing questionnaire items used for scoring were set to the baseline value of zero (items 3 to 10) for the primary approach. Random imputation of missing questions/answer categories (eg question 6, 8) in the validation cohort used frequency estimates from the development dataset. For random imputation of the total score we reshuffled the validation cohort's actual scores between individuals. Both imputations were run 100 times, measures of performance averaged, and in-between imputation variation accounted for the calculation of confidence intervals. These approaches will not add further insight to the performance of the score, but allows assessment of robustness. Reshuffling the scores randomly gives non-informative point and precision estimates, facilitating interpretation of real performance measures. The actual score distribution in the development cohort was inferred from tabulated sensitivity and specificity, as it was not published in the original article.

A univariate logistic model was used to derive measures of test performance (sensitivity, specificity, predictive values, likelihood ratios) and disease probability for each score, resembling what was reported originally. Odds ratio for developing asthma per 1-point increase of the score at 3 years as well as Nagelkerke's R^2^ (maximum rescaled R^2^, coefficient of determination standardized to its maximum) and maximum rescaled Brier score were calculated as overall performance measures [24]. The Brier score is a measure of how well the predicted and the actual outcomes overlap: with a value of 0 the model adds no information to the a-priori prevalence, and a value of 1 indicates best-possible prediction. Discrimination was reported using the c statistic (AUC, area under curve), calibration/agreement was assessed graphically plotting predicted disease frequency in eight groups against observed disease frequency.

## Results

### Study population

Our sample from the MAS-90 cohort is similar to the cohort population the score was developed in, with respect to prospective regular assessments. Unlike in the original cohort from Leicester, UK, where children were enrolled aged 1 to 3, the German MAS-90 participants were all recruited at birth and allergic parents were slightly oversampled (table 1).

**Table 1:** Comparison of study characteristics.

	Development cohort	External validation cohort
Location	Leicester (United Kingdom)	Berlin, Düsseldorf, Freiburg, Mainz, Munich (Germany)
Climate	humid temperate maritime	humid temperate maritime/continental
Year of birth	1995–97	1990
Male sex	52%	52%
Study design	prospective cohort (birth and later)	prospective birth cohort
Recruitment	general population random sample	population-based (partly risk-enriched regarding parental allergy)
Ethnicity (mother)	81% Caucasian 19% south Asian	ethnicity unknown, predominantly Caucasian
Age at scoring (median)	2 years	3 years

841 of 1,314 (64%) study participants completed follow-up assessments at 3 and 8 years, of which 140 (17%) met the primary inclusion criteria: wheeze or cough in the previous 12 months at age 3 years. This primary study sample was similar to all followed, in terms of parental education and overweight, family's smoking habits, and atopic heredity (parents' self-reported allergies and cord blood Immunoglobulin E, table 2). 121 children from the primary sample (86%) were successfully traced up to the age of 20 years, out of which 93 (77%) underwent lung function testing.

**Table 2:** Characteristics of families in sample used for external validation, by follow-up status.

		assessed at 3 and 8y		loss to follow-up		met primary inclusion criteria	
		n	% [95%-CI]	n	% [95%-CI]	n	% [95%-CI]
All		841	100	473	100	140	100
Parental educational level							
	low (ISCED 1/2)	143	18 [16]; [20]	122	28 [24;32]	26	20 [12]; [28]
	medium (ISCED 3/4)	337	43 [39;46]	187	43 [39;47]	52	40 [32;49]
	high (ISCED 5/6)	312	39 [36;43]	128	29 [25;32]	51	40 [33;48]
Overweight (BMI ≥25 kg/m2)							
	mother	122	18 [15]; [21]	35	25 [17;31]	15	14 [7]; [19]
	father	280	44 [40;49]	58	48 [38;58]	38	40 [33;48]
Smoking							
	at home	439	56 [53;60]	289	82 [78;86]	73	55 [48;61]
	during pregnancy	89	11 [10]; [14]	82	19 [15]; [23]	18	13 [9]; [18]
Asthma, allergic rhinitis or eczema							
	parents not allergic	412	50 [45;54]	252	55 [51;59]	55	40 [32;47]
	mother or father	324	39 [36;44]	154	34 [30;38]	61	45 [38;52]
	both parents	87	11 [8]; [13]	52	11 [7]; [13]	21	15 [9]; [22]
Cord blood IgE ≥0.35 kU/l		288	36 [32;39]	163	36 [31;39]	56	43 [36;50]

ISCED, International Standard classification of Education; BMI, Body mass index; IgE, Immunoglobulin E; 95%-CI, bootstrapped 95% confidence intervals; kU/l, kilo Units per litre.

### Scoring

We identified corresponding items in the 3-year-questionnaire for 9 of 10 original scoring questions, 5 with perfect/very good and 1 with good comparability. One question (item 5, wheeze interfering with daily activities) was not asked in the validation cohort, but could be substituted by a proxy question on sleep disturbance by wheeze. One question (item 8, cause of wheeze/cough) could not be replaced by a meaningful alternative, which was assessed in sensitivity analyses as described later (table 3).

**Table 3:** Questionnaire items used for scoring.

Question in asthma prediction score [13]			(%)	Questionnaire item in MAS-90 (3y-follow-up)		(%)	Comparability
1.	What is the child's sex?	Female = 0	(45)	[assessed at baseline]	Female = 0	(46)	perfect
		Male = 1	(55)		Male = 1	(54)	
2.	How old is the child? (in years)	1 = 0	(28)	[calculated from date of birth and date at 3y-follow-up]	-	-	Perfect (limited to 3y)
		2 = 1	(57)		-	-	
		3 = 1	(15)		3 = 1	(100)	
3.	In the last 12 months, has the child had wheezing or whistling in the chest even without having a cold or flu?	No = 0	(82)	Did this wheezing or whistling go along with a cold or flu?	Always/Don't know = 0	(83)	very good (negation of question)
		Yes = 1	(18)		No/Sometimes = 1	(17)	
4.	How many attacks of wheeze has the child had during the last 12 months?	0-3 = 0	(45)	How many attacks of wheezing or asthma has your child had during the last 12 months?	0-3 = 0	(47)	very good (asthma included)
		>3 = 2	(55)		>3 (3 cat.) = 2	(53)	
5.	In the last 12 months, how much did wheezing interfere with your child's daily activities?	Never = 0	(64)	In the last 12 months, how often, on average, has your child's sleep been disturbed due to wheezing or asthma?	Never = 0	(67)	Moderate (proxy measure of severity)
		A little = 1	(26)		Only at attacks = 1	(26)	
		A lot = 2	(10)		Regularly (3 cat.) = 2	(7)	
6.	Do these wheezing attacks cause him/her to be short of breath?	Never = 0	(65)	In the last 12 months, was an attack of wheezing or asthma severe enough that your child had to take a breath after one or two words?	No = 0	(84)	Moderate (answer category missing)
		Sometimes = 2	(29)		Yes = 2	(16)	
		Always = 3	(6)		-	-	
7.	In the last 12 months, did exercise (playing, running) or laughing, crying or excitement cause wheezing or coughing in the child?	No = 0	(61)	In the last 12 months, did physical stress cause wheezing in the child?	No = 0	(81)	Good (cough not included, different answers not listed)
		Yes = 1	(39)		Yes = 1	(19)	
8.	In the last 12 months, did contact with dust, grass, pets or other animals cause wheezing or coughing in the child?	No = 0	(93)	[no comparable item available]			-
		Yes = 1	(7)				
9.	Has the child ever had eczema?	No = 0	(56)	In the last 12 months, has your child had eczema?	No = 0	(63/25*)	very good (last 12 months vs. ever)
		Yes = 1	(44)		Yes = 1	(37/75*)	
10.	Have the child's parents ever suffered from wheezing, asthma or bronchitis?	None = 0	(52)	In the last 12 months, did the child's mother/father suffer from asthma?	No = 0	(86/48*)	Moderate (wheezing/bronchitis not included, last 12 months vs. ever)
		Mother = 1	(22)		Mother = 1	(8/34*)	
		Father = 1	(17)		Father = 1	(6/29*)	

Numbers in brackets (%) are frequencies of parents' answers, denominator of n = 1226 in Leicester subsample used for generating the tool, and of n = 140 in MAS-90 subsample. * before/after imputation with values from prior follow-up assessments and baseline.

Frequency of answers was similar in the validation cohort compared to the original population for most items, except for shortness of breath at 16% vs. 35% in development cohort and wheezing caused by ‘physical stress’ at 19% vs. 39% in development cohort, which asked for wheezing caused by ‘exercise, laughing, crying or excitement’. Furthermore, parents' respiratory illness was more common in the Leicester population asking for wheeze, asthma and bronchitis (mother 22%/father 17%), compared to MAS-90 where the question was limited to asthma only (8%/6%).

**Figure 1:**
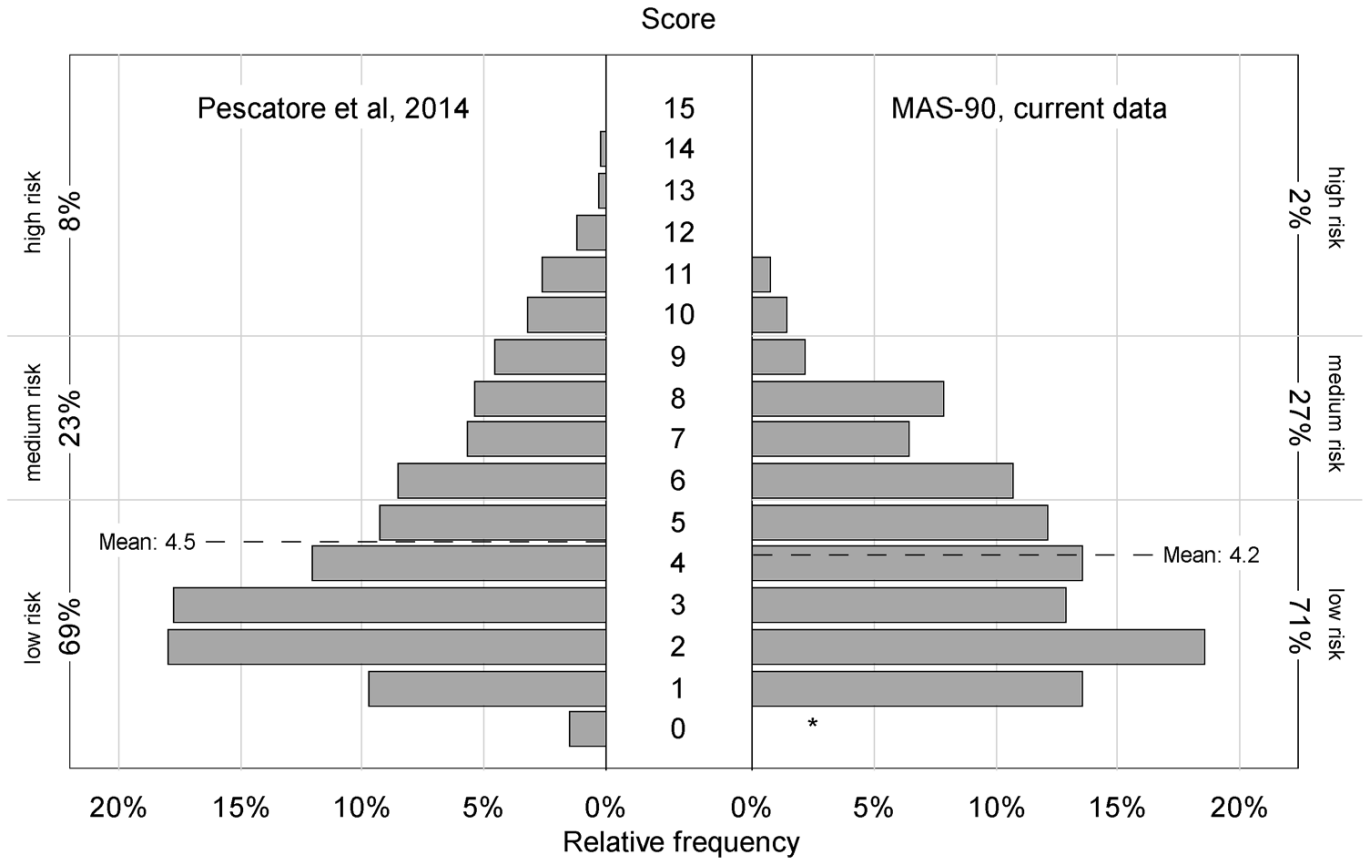
Score distribution (relative frequency) from model building sample. (n = 1226, left side, from [13]) and external validation sample (n = 140, right side). Primary inclusion, scoring and outcome definitions. * no child with age 1 year in external validation sample (score 0 not possible).

The distribution of actual scores in the validation cohort (mean score 4.2, median 4) was very similar to what we derived from the report of Pescatore et al. (mean score 4.5, median 4). Only the high risk group (score ≥10) was considerably smaller in our sample with 2% vs. 8% (Fig. 1).

### Outcomes

28 of 140 children (20%) who wheezed or coughed at 3 years of age met the primary outcome definition of asthma at 8 years (wheeze combined and asthma medication, both parent-reported). Regarding specific symptoms, 33 (26%) reported wheeze, 33 (26%) recent use of asthma drugs, and 27 (19%) a physician's diagnosis of asthma. 51 of 121 children (42%) followed up to 20 years developed asthma between 3 and 20 years, the majority was sensitized to aero-allergens (84%). 29 of 93 (31%) participants at age 20 reacted to Methacholine challenge in lung function testing. In the low risk group (score ≤5) asthma prevalence at 8 years was 9% (vs. 16% in original cohort), in those at medium risk (score 6–9) 45% (vs. 48%). Items used for inclusion and the primary outcome definition were comparable between the original and the validation cohort (table 4).

**Table 4:** Questionnaire items used as inclusion criteria and outcome definition, comparing original and validation cohort.

Leicester cohort: Items for inclusion criteria (at age 1-3 years)		MAS-90 cohort: Items for inclusion criteria (at age 3 years)		Comparability
Has your child had wheezing or whistling in the chest in the last 12 months?	yes, no	Has your child had wheezing or whistling in the chest in the last 12 months?	yes, no	perfect
Does your child usually have a cough apart from colds?	yes, no	[no comparable item available]	-	undefined use in [13]
In the last 12 months, has your child had a dry cough at night, apart from a cough associated with a cold or a chest infection?	yes, no	In the last 12 months, has your child had a dry cough at night, apart from a cough associated with a cold or a chest infection?	yes, no	perfect
How often did your child see a GP for coughing or wheezing during the last 12 months?	never, once, 2–3 times, 4–6 times, 7 or more times	[no comparable item available]	-	-
In the last 12 months, has wheezing or asthma resulted in your child: [4 categories: referred/admitted to hospital, attending/calling ER or GP]	yes, no	[no comparable item available]	-	undefined use in [13]
Leicester cohort: Items for outcome definition (at age 8 years)		MAS-90 cohort: Items for outcome definition (at age 8 years)		
Has your child had wheezing or whistling in the chest in the last 12 months?	yes, no	Has your child had wheezing or whistling in the chest in the last 12 months?	yes, no	perfect
Did your child take any of the following during the last 12 months? [4 categories: inhalers by content/type]	yes, no, don't know	Did your child take any drugs (syrup, tablets or spray) against breathing difficulties during the last 12 months?	yes, no	Moderate (no details on drugs taken)

### Model performance

**Figure 2:**
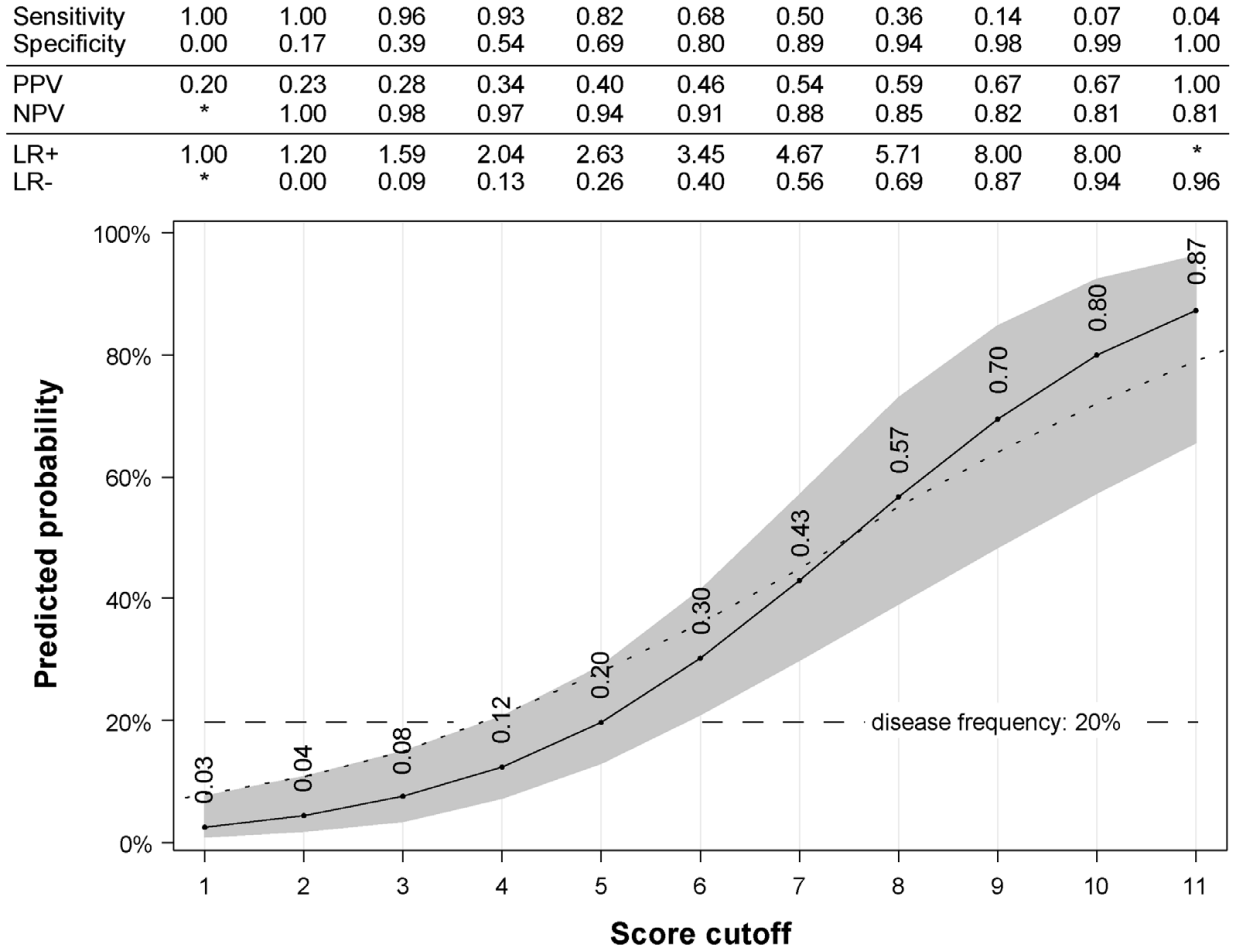
Predicted probability of developing asthma 5 years later, for a single particular score value. Performance measures by score in table above. Primary inclusion, scoring, and outcome definitions. Probabilities from original paper (dotted line). * Values not meaningful and/or no accurate estimate possible. PPV/NPV, positive/negative predictive value; LR+/-, positive/negative likelihood ratio.

**Figure 3:**
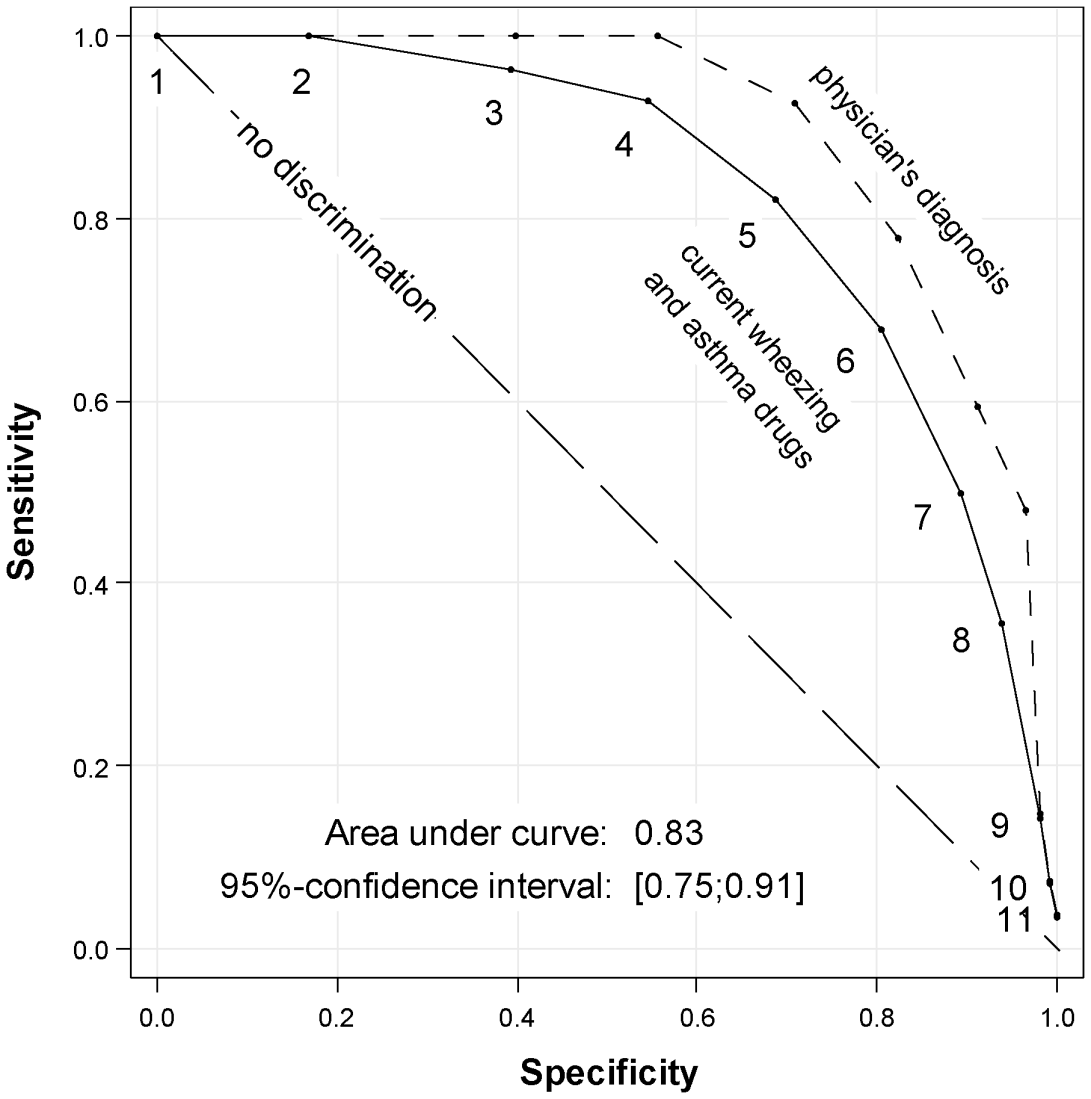
Receiver Operating Characteristic (ROC) using external validation sample. Primary inclusion and scoring definitions. Outcome definitions: Primary (current wheezing and asthma drugs at 8y, solid line) and secondary (physician's diagnosed asthma at 8y, dashed line). Numbers indicate asthma prediction score values. The area under the curve (AUC) and confidence interval corresponds to the primary outcome.

**Figure 4:**
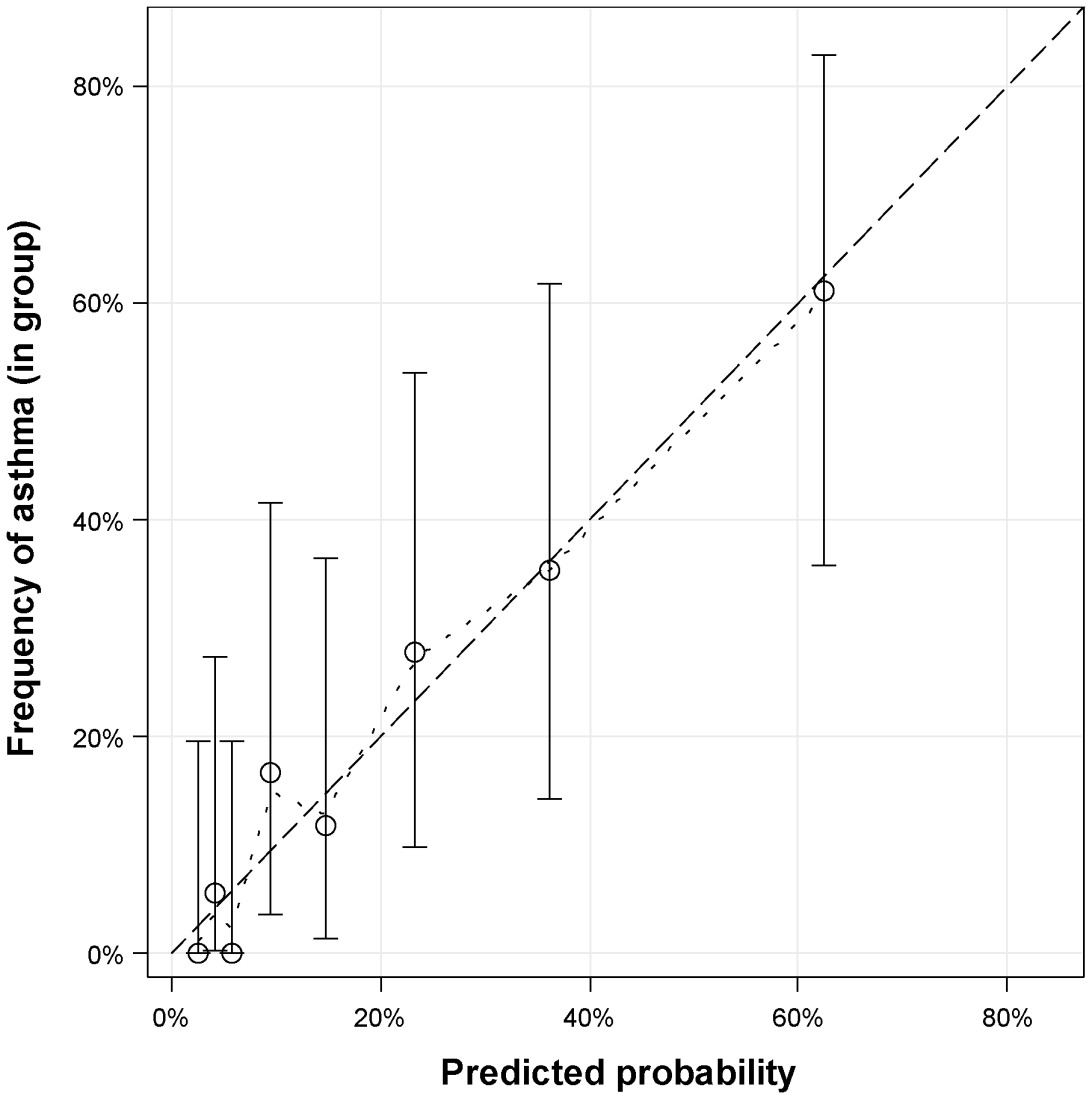
Calibration assessment, predicted probabilities vs. real asthma frequencies in eight equally assigned groups. Exact 95%-confidence intervals for actual asthma frequency based on beta/binominal distribution. Penalized B-Spline (2^nd^ degree) aiding graphical interpolation (dotted line). Perfect calibration at diagonal (dashed line). Primary inclusion, scoring, and outcome definitions.

Performance of the primary model in our sample was similar to the original cohort with sensitivity of 82% (original cohort 72%) and specificity of 69% (original cohort 71%) at score 5. Predicted disease probability at score 5 was 20%, by chance the same as the a priori disease frequency (Fig. 2). Overall performance of the primary model resembled the original analysis with a max-rescaled Brier score of 0.22, Nagelkerke's R^2^ (max-rescaled) of 0.32 and an odds ratio of 1.7 (95% confidence interval 1.4–2.1). Discrimination between asthma vs. no asthma at age 8 years was better in our sample with an AUC (area under the curve) of 0.83 (95% confidence interval 0.75–0.91), compared to 0.78 in the Leicester sample. Replacing the primary case definition with a physician's diagnosis of asthma led to an even higher AUC of 0.89 (Fig. 3). Graphical assessment of agreement between observed and predicted disease frequencies revealed very good calibration of the original model (Fig. 4).

### Sensitivity analyses

We reiterated discrimination and performance assessment for various inclusion, scoring and outcome criteria. The model performed best using cough as the only inclusion criterion at 3 years (AUC 0.91, Brier score 0.45) or a physician's diagnosis of asthma as outcome definition at 8 years (AUC 0.89, Brier score 0.34). The model performed poorly in predicting response to the Methacholine challenge at 20 years (AUC 0.64, Brier score 0.05). Randomly reshuffled scores showed, expectedly, no discrimination and prediction (table 5).

**Table 5:** Discrimination and Performance of the asthma prediction score in the MAS-90 birth cohort used for external validation.

	n	Cases^$^	(%)	Area under curve [95%-CI]		Nagelkerke's R^2^ *	Brier score*	Odds Ratio [95%-CI]	
**Primary inclusion, scoring, and outcome^§^**	140	28	(20)	0.83	[0.75;0.91]	0.32	0.22	1.7	[1.4;2.1]
**Secondary inclusion definitions**									
all available	841	48	(6)	0.80	[0.73;0.87]	0.26	0.18	1.8	[1.6;2.1]
non-allergic parents	412	9	(2)	0.84	[0.70;0.97]	0.26	0.12	2.0	[1.5;2.7]
wheezing	81	26	(32)	0.72	[0.61;0.84]	0.18	0.13	1.5	[1.2;2.0]
cough	80	13	(16)	0.91	[0.81;1.00]	0.55	0.45	2.1	[1.5;3.1]
**Secondary scoring definitions**									
complement from earlier follow-ups	140	28	(20)	0.83	[0.75;0.90]	0.33	0.21	1.8	[1.4;2.2]
missing answers filled at random^#^	140	28	(20)	0.83	[0.75;0.91]	0.33	0.23	1.7	[1.4;2.2]
actual scores randomly shuffled^#^	140	28	(20)	0.55	[0.41;0.69]	0.01	0.01	1.0	[0.8;1.3]
**Secondary outcome definitions**									
Wheeze at 8y	140	33	(24)	0.81	[0.73;0.89]	0.30	0.21	1.6	[1.3;2.0]
asthma drugs at 8y	140	33	(24)	0.82	[0.74;0.90]	0.32	0.23	1.7	[1.4;2.0]
physician's diagnosis asthma (ever)	140	27	(19)	0.89	[0.84;0.95]	0.47	0.34	2.1	[1.6;2.7]
**Secondary outcome definitions (later)**									
any asthma up to 20y	121	51	(42)	0.75	[0.66;0.84]	0.24	0.20	1.5	[1.3;1.8]
allergic asthma up to 20y	121	43	(36)	0.80	[0.72;0.88]	0.31	0.24	1.6	[1.3;2.0]
wheeze/asthma medication at 13y	113	14	(12)	0.86	[0.78;0.95]	0.36	0.23	2.0	[1.4;2.9]
wheeze/asthma medication at 20y	121	18	(15)	0.80	[0.68;0.91]	0.27	0.19	1.6	[1.3;2.1]
increased airway responsiveness at 20y	93	29	(31)	0.64	[0.52;0.77]	0.07	0.05	0.8	[0.7;1.0]

* rescaled to its maximum value; # 100 imputations, accounted for calculating confidence intervals; $ according to the corresponding outcome definition; § primary inclusion: cough or wheeze at 3 years – primary scoring: available items at 3 years – primary outcome: wheeze and asthma medication at 8 years; Odds Ratio per 1-point increase in asthma prediction score; Secondary outcome definition details up to age 20 years in methods section; CI, confidence interval.

## Discussion

### Key results

We externally validated the recently developed asthma prediction tool [13] retrospectively on follow-up data of the German MAS-90 birth cohort study, which resembles major design aspects of the original cohort from Leicester, UK [20]. 9 of 10 scoring items were successfully mapped to our questionnaire at 3 years of age, with similar answer frequencies compared to the development cohort. The final score distribution and asthma frequencies at 8 years within low (9% reported asthma), middle (45%) and high risk (67%) score groups were close to the original sample. Measures of performance were similar (max-rescaled Brier score 0.22, max-rescaled/Nagelkerke's R^2^ 0.32) and discrimination slightly better (AUC 0.83) compared to the Leicester sample (AUC 0.78). Sensitivity analyses revealed robust prediction for various definitions of inclusion, scoring and outcome criteria, with even better discrimination using the stringent outcome definition of asthma diagnosed by a physician (AUC 0.89).

### Strengths and limitations

This retrospective validation analysis was made possible because follow-up assessments in the MAS-90 cohort included all data necessary to derive inclusion criteria, information for scoring according to the ten original asthma prediction tool questions, and outcome definitions. Such rare opportunity is an ideal setting for external validation: a similar population unrelated in terms of location and sampling, assessed with the same or similar tools [15].

In the original cohort inclusion was based on the report of wheeze or cough at 3 years, for the assessment at 2 years (median age of original cohort) did not include these and other information necessary for scoring, and used the exact same questionnaire wording as we did in our birth cohort. The original sample was further limited to those having recently seen a physician in response to these symptoms. Such information was not collected in the MAS-90 cohort and could not be replaced by proxy data. But model performance and discrimination was similar applying different (secondary) inclusion criteria, with the subsample of children reporting wheeze giving the poorest discrimination (AUC 0.72) and those with cough the highest (AUC 0.91). Compared to recommended sample sizes for validation studies, the validation sample yielded a limited number of events and non-events [27], with slightly less precise estimates in the validation cohort.

Only little information necessary to calculate the exact score for each child was missing in our sample, either because it was missing by item or the according question was not asked or phrased differently. We approached the latter by mapping related questionnaire items containing proxy information. The two secondary definitions used for scoring gave almost identical performance and discrimination, one complementing data from earlier follow-up assessments, the other iteratively imputing missing items with random values. The minor difference in risk distribution with lower numbers for the highest scores in our sample and the predominantly Caucasian population limit generalizability of this validation. The different age distributions at risk assessment including 1–2 years old children in the original cohort may explain the lower performance measures. Of note, prevalence and severity of asthma–like symptoms are higher in the United Kingdom compared to Germany [28].

Wheezing in the past 12 months along with the use of asthma inhalers at 8 years was the outcome used for model development. Our 8-years' questionnaire asked non-specifically for any drugs against respiratory disease including those administered orally, leading to wider inclusion. A noteworthy feature of our analysis was the assessment of various (secondary) outcome definitions. Those criteria based solely on information from the 8-years' questionnaire gave similar performance and discrimination with the highest AUC of 0.89 for predicting asthma supported by a physician's diagnosis at 8 years. The asthma prediction tool performed worse predicting any asthma up to age 20 years (AUC 0.75), allergic asthma up to 20 years (AUC 0.80) and did not predict airway responsiveness at 20 years (AUC 0.64).

### Appraisal

As expected for retrospective external validation, there was no perfect resemblance of tools and criteria. But robustness of performance and discrimination against changes of inclusion, scoring and outcome criteria gave confidence for the applicability of the asthma prediction tool in settings outside the development cohort.

Prediction usually performs best in the setting where the model is developed in, either internally on the same or a subset of the original sample, or externally in the same setting shifted geographically and/or in time [16], [18]. To our surprise, the external validation that we performed in a different setting, the MAS-90 birth cohort from Germany, gave even better performance, discrimination and prediction. This could be due to differences in questionnaire administration: filled by parents in the original cohort vs. based on face-to-face interviews in MAS-90, the latter presumably with higher discriminatory power. Additionally, the LASSO method (Least Absolute Shrinkage and Selection Operator) used for predictor selection while developing the score aims at fewer factors in the final model and higher external validity [29].

### A single prediction tool

Several independent attempts to predict asthma development from preschool symptoms and presumed risk factors have been made, but none reached widespread application in clinical practice [9]. Most tools were never validated externally, never updated and refined further, and their health benefit never assessed. Instead of incrementally improving existing prediction tools, new but similar models with comparable sets of predictors were suggested.

Validating this tool externally, we see our analysis as a first step towards a single and robust tool for the prediction of asthma development. This should be done in other cohorts retrospectively, and ongoing or future longitudinal research on asthma should consider collecting information detailed enough to facilitate external validation of existing prediction tools. Comparing performance between the model development cohort and various external settings provides valuable insight into the influence of sampling and inclusion, selectiveness of scoring information and outcome criteria.

From this general understanding, the existing model should be updated. This includes the identification of additional factors not yet incorporated, and the refining of weights from re-running the model. As the search for risk-factors has a focus on true modifiable causes of the disease, this is not the case for estimating the individual probability to develop asthma. Non-modifiable traits such as parents' allergy status, indicators of pre-clinical disease (eg, early wheezing), sensitization, and environmental as well as behavioural exposures provide valuable information for prediction modelling and should be used to improve the model further.

Re-weighting prediction factors in external settings is often hampered by the lack of detailed descriptions of the model building process, which should always be made available online along initial publications, just as done in Pescatore et al. [13]. In-depth sensitivity analyses are inevitable for robust re-weighting of parameter estimates, accounting for inclusion, score coding and different outcomes.

As a final step, impact analyses are essential to predict health benefits of applying asthma prediction tools in clinical practice. They could prove useful for early interventions, targeting prevention strategies, or improve sampling strategies for research.

### Conclusion

This first external validation of the newly developed asthma prediction tool supports applicability outside the development cohort from Leicester, UK. Performance and discrimination were better in our external sample compared to internal validation, and robust against changes of inclusion, scoring and outcome definitions. Being a tool for timely diagnosis based primarily on early symptoms, we support its development by incorporating risk factors, externally refining the underlying model and assessing the impact on health outcomes.
